# Endoscopic injection sclerotherapy for pediatric bleeding esophageal varices complicated by gastric vein, main portal vein, splenic mesenteric junction, and splenic vein occlusion: a case report

**DOI:** 10.1186/s12876-019-0955-7

**Published:** 2019-02-28

**Authors:** King-Wah Chiu, Ting-Lung Lin, Chee-Chien Yong, Chih-Che Lin, Yu-Fan Cheng, Chao-Long Chen

**Affiliations:** 1 Chang Gung University, College of Medicine, Taoyuan, Taiwan; 2 Liver Transplant Program, Kaohsiung Chang Gung Memorial Hospital, Kaohsiung, Taiwan; 3 Department of Internal Medicine, Kaohsiung Chang Gung Memorial Hospital, Kaohsiung, Taiwan; 4 Department of General Surgery, Kaohsiung Chang Gung Memorial Hospital, Kaohsiung, Taiwan; 5 Department of Diagnostic Radiology, Kaohsiung Chang Gung Memorial Hospital, Kaohsiung, Taiwan

**Keywords:** Biliary atresia, Complication, Endoscopic injection sclerotherapy, Esophageal varices, Living donor liver transplantation

## Abstract

**Background:**

Endoscopic injection sclerotherapy (EIS) is a life-saving procedure for pediatric patients with bleeding gastric varices (GV) associated with advanced liver cirrhosis and severe portal hypertension. Because of the lack of an endoscopic banding ligation device for pediatric patients, EIS is usually performed for bleeding esophageal varices (EV) in infants with congenital biliary atresia.

**Case presentation:**

We present a case of a 15-month-old female infant with type I biliary atresia with jaundice (total serum bilirubin, 22.2 mg/dL), hypoalbuminemia (serum albumin level, 2.58 g/dL), coagulopathy (prothrombin time > 20 s compared with that of a normal control), ascites, splenomegaly, portal hypertension (portal vein velocity, 3.9–5.6 cm/sec of hepatopetal flow), and repeated bleeding of the varices after receiving three doses of intravascularly administered Histoacryl 1 ampoule mixed with Lipiodol UF 8 mL in the EV. Prominent GV and EV were occluded by EIS. The sclerosing agent was also present in the main portal vein, splenic mesenteric junction, and splenic vein, causing an engorged inferior mesenteric vein. The patient underwent total hepatectomy and living donor liver transplantation (LDLT) by left lateral segment graft (segments 2, 3, and 4 of the middle hepatic vein trunk) and left portal vein graft to the recipient inferior mesenteric vein anastomosis. Portal vein stent placement via segment 4 of the portal vein stump was performed from the inferior mesenteric vein to the umbilical portion of the left portal vein. The patient is still alive and doing well after the LDLT.

**Conclusions:**

EIS is a life-saving procedure in cases involving bleeding EV complicated by gastric, main portal vein, splenic mesenteric junction, and splenic vein occlusions; hence, it should be kept in mind as a treatment for EV complications in pediatric patients.

## Background

Endoscopic hemostasis for bleeding esophageal varices (EV) is widely used along with endoscopic banding ligation. In contrast, endoscopic injection sclerotherapy (EIS) is the treatment of choice for bleeding gastric varices (GV) [1, 2]. Because the devices for banding ligation are only suitable for adult patients, EIS is also a life-saving endoscopic hemostatic treatment for pediatric patients with bleeding EV and/or GV. We present a case of a pediatric patient with a rare complication treated with EIS.

## Case presentation

**Fig. 1:**
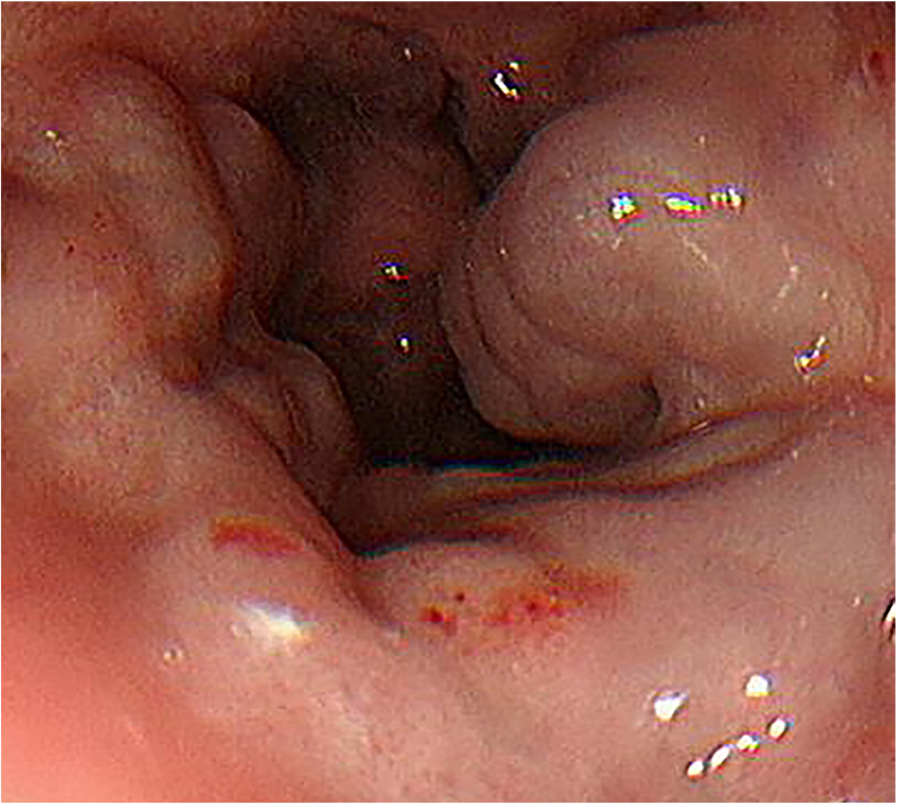
Endoscopic picture of severe esophageal varices

**Fig. 2:**
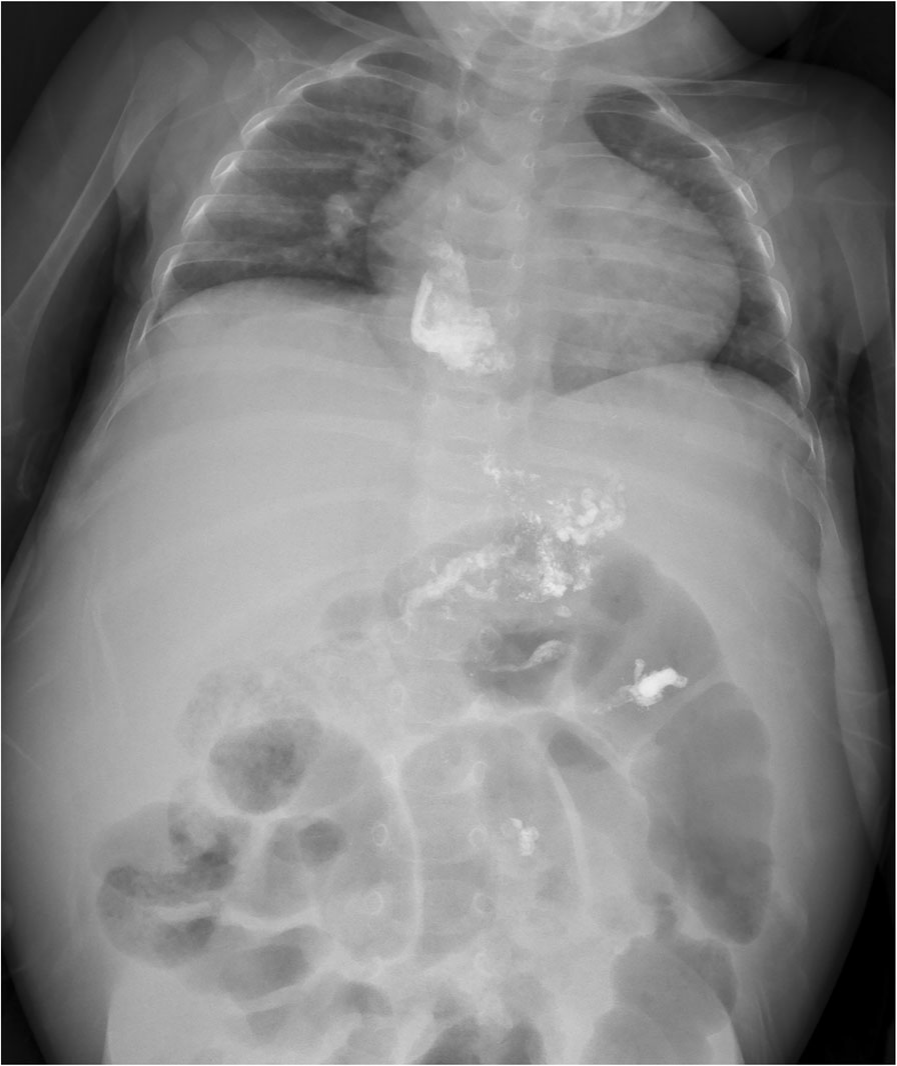
Plant X-ray film showing that the sclerosing agent was occluded in the esophageal and gastric varices and extended into the splenic vein and part of the superior mesenteric veins

**Fig. 3:**
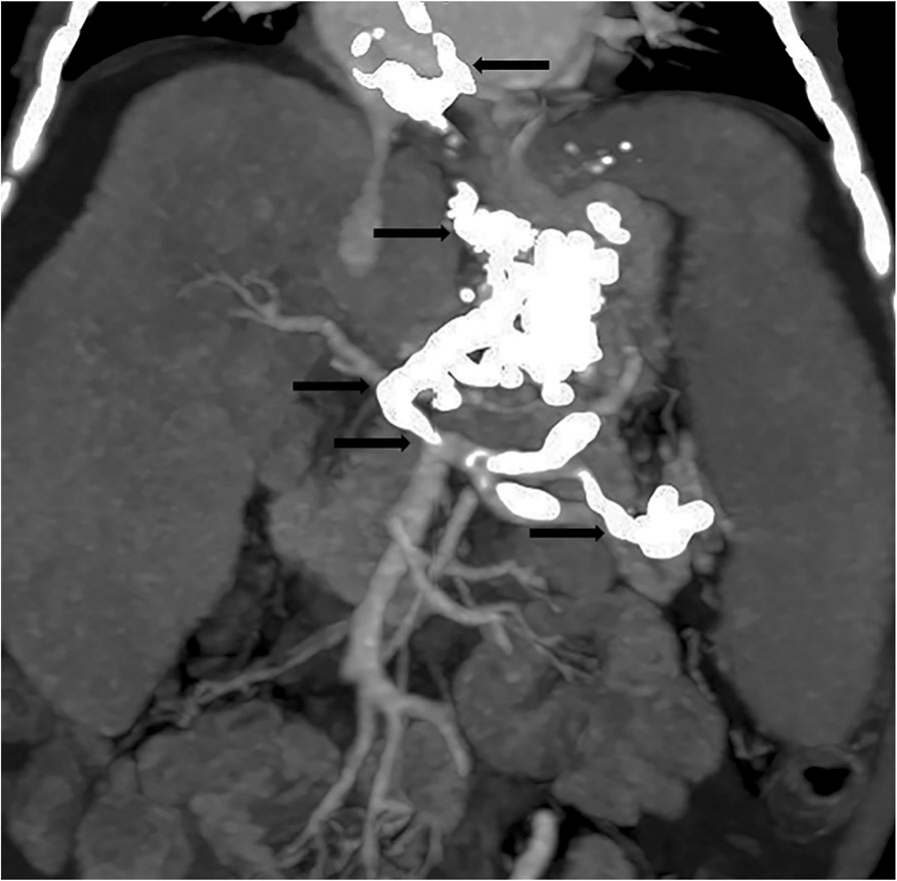
Abdominal computed tomography showing that the sclerosing agent was occluded in the esophageal varices, gastric varices, main portal vein, splenic mesenteric junction, and splenic veins (black arrows) and engorged the inferior mesenteric vein

**Fig. 4:**
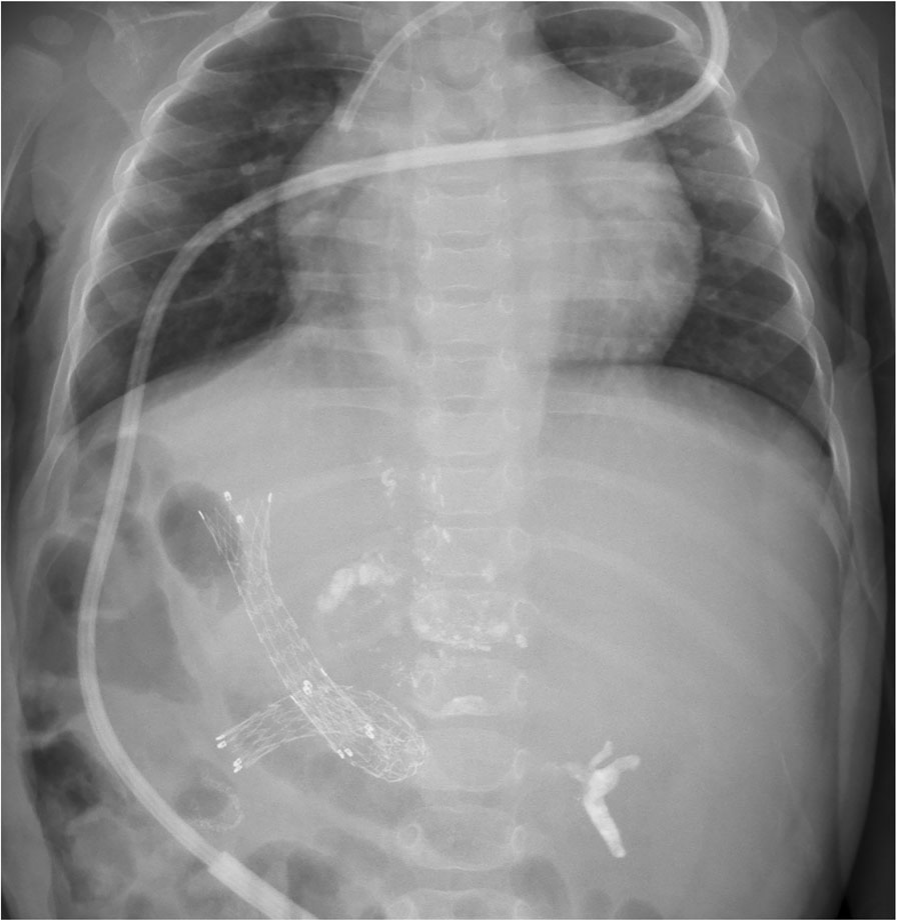
Portal vein stent placement via segment 4 of the portal vein stump was performed from the inferior mesenteric vein to the umbilical portion of the left portal vein

A 15-month-old female Filipino infant with congenital type I biliary atresia and without any other anomalies or malformations, who had not undergone Kasai’s surgical procedure for biliary atresia, was referred by a liver center in the Philippines. She weighed 8.1 kg and had a height of 67.3 cm. She had jaundice (total serum bilirubin, 22.2 mg/dL), hypoalbuminemia (serum albumin level, 2.58 g/dL), coagulopathy (prothrombin time > 20 s compared to that of a normal control), ascites, splenomegaly, portal hypertension (portal vein velocity, 3.9–5.6 cm/sec with hepatopetal flow measured by Doppler ultrasound), and repeated bleeding of the varices after three doses of intravascularly administered Histoacryl 1 ampoule mixed with Lipiodol UF 8 mL (Auckland, New Zealand) in the EV (Fig. 1). A Doppler ultrasound was used to investigate the portal hemodynamics before EIS. The diameter of the portal vein was 6.1 mm with reversal hepatofugal flow in portal vein velocity. After the first EIS, the portal vein diameter was 4.4 mm without thrombosis. After the third EIS, the end point of EIS was further investigated, and computed tomography angiogram revealed that the intrahepatic portion of the portal vein was not clearly demonstrated. Prominent GV and EV were occluded by EIS (Fig. 2). The sclerosing agent was not only present in the EV and GV but also retrogradely occluded the main portal vein, splenic mesenteric junction, and splenic vein, causing an engorged inferior mesenteric vein (Fig. 3). The patient underwent total hepatectomy and living donor liver transplantation (LDLT) via a left lateral segment graft (segments 2, 3, and 4 of the middle hepatic vein trunk) and left portal vein graft for the recipient inferior mesenteric vein anastomosis. Portal vein stent placement via segment 4 of the portal vein stump was performed from the inferior mesenteric vein to the umbilical portion of the left portal vein (Fig. 4). The patient is still alive and doing well after the LDLT.

## Discussion and conclusions

**Fig. 5:**
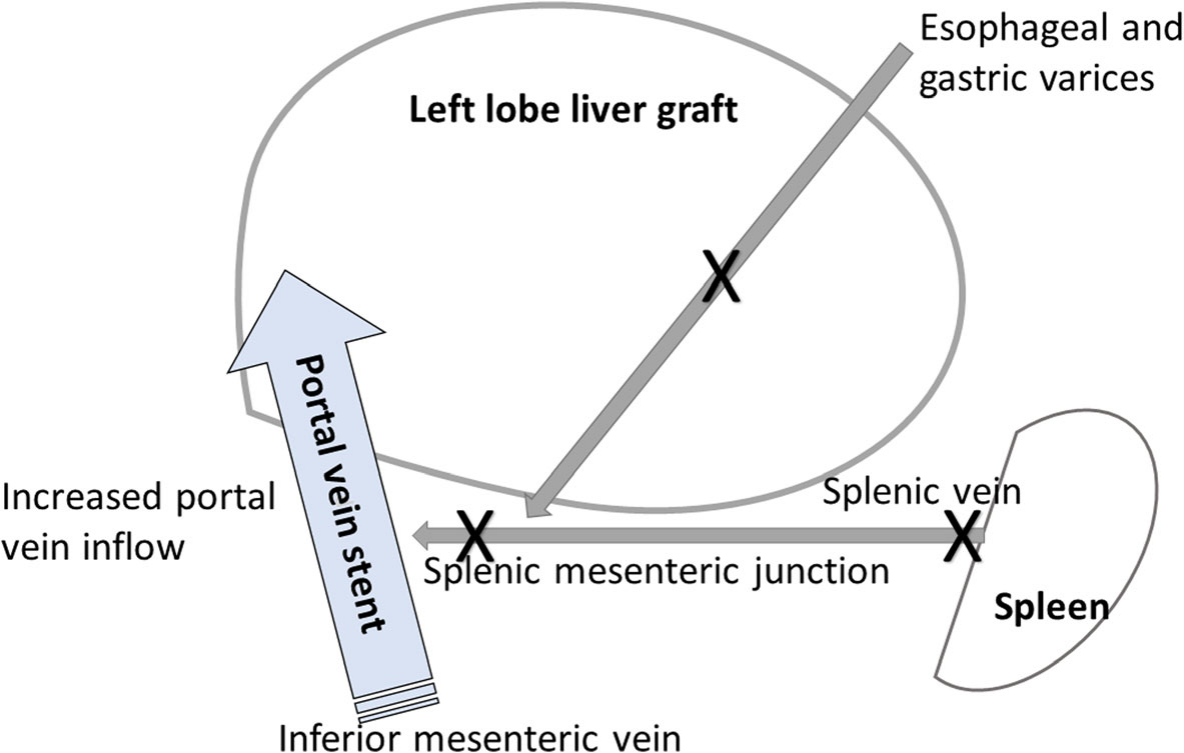
Schematic picture of the portal systems and the direction of blood flow for the portal vein stent with portal vein and inferior mesenteric vein anastomosis. The **X** means the insufficient blood inflow from the esophageal and gastric varices, splenic and superior mesenteric veins to the portal vein compromised the liver graft. The big arrow means a portal vein stent was placed to increase the inferior mesenteric vein size and augment the portal vein flow to ensure sufficient blood flow to the portal system of the new liver graft

Thrombosis in the portal, splenic, and mesenteric veins may develop after sclerotherapy in cirrhotic patients [3, 4]. This case is interesting because it involves an infant. The insertion tube outer diameter of adult and pediatric gastroscope is 9.0–11.4 and 5.9–6.0 mm, respectively. The cap of the ligation device can only hold tightly on a gastroscope with outer diameter > 10–11.4 mm; however, such a diameter is hard to insert to a 15-month infant patient with an esophageal orifice diameter < 10 mm. Therefore, ligation of varices was only performed in older pediatric or adult patients with an esophageal orifice diameter > 10 mm. EIS was frequently used to stop bleeding in infants or those with esophageal orifice diameter < 10 mm. In fact, EIS rarely induces gastric, main portal vein, splenic mesenteric junction, and splenic vein occlusion in pediatric patients. The dose of the intravascularly administered Histoacryl mixed with Lipiodol differs between adult and pediatric patients because the size of the vascular structures is smaller in the latter. According to the current presentation, half a dose of the sclerosing agent, i.e., Histoacryl 1/2 ampoule mixed with Lipiodol 4 mL, is suggested for the treatment of bleeding varices in pediatric cases. LDLT should be the treatment of choice not only for pediatric patients with biliary atresia but also for correcting complications arising from EIS with regards to splenic and superior mesenteric vein occlusion [5]. In our case, the sclerosing agent occluded the splenic and portal veins and retrogradely down to the splenic mesenteric junction. We placed a portal vein stent to avoid thrombosis of the mesenteric vein in this patient who underwent LDLT. Because the portal vein flow was low (9.8 cm/sec) and the inferior mesenteric vein was small during portal vein anastomosis, we also worried about the insufficient blood inflow from the splenic and superior mesenteric veins to the portal vein and compromised liver graft. A portal vein stent was placed to increase the inferior mesenteric vein size and augment the portal vein flow to ensure sufficient blood flow to the portal system of the new liver graft (Fig. 5). The portal vein stent placement via segment 4 stump approach for intraoperative portal vein stenting in pediatric LDLT is an innovative technique for this kind of complication. Hence, we would like to share our experience and contribute to the medical field. In conclusion, endoscopic hemostasis with EIS is a life-saving procedure not only for bleeding EV but also for the treatment-associated major complications with gastric vein, main portal vein, splenic mesenteric junction, and splenic vein occlusions in pediatric patients with biliary atresia.

## Abbreviations

EIS: Endoscopic injection sclerotherapy
EV: Esophageal varices
GV: Gastric varices
LDLT: Living-donor liver transplantation
